# P-2141. Factors Associated With Death in a Population-based Invasive Mold Surveillance Program, Atlanta, GA

**DOI:** 10.1093/ofid/ofae631.2296

**Published:** 2025-01-29

**Authors:** Nora T Oliver, Carolyn Mackey, Dana Goodenough, Stepy Thomas, Lucy S Witt

**Affiliations:** Atlanta VA Medical Center, Decatur, Georgia; Georgia Emerging Infections Program, Atlanta, Georgia; Emory University School of Medicine, Atlanta, Georgia; Emory University, Atlanta, Georgia; Emory University, Atlanta, Georgia

## Abstract

**Background:**

Invasive mold infections (IMI) can lead to severe illness and are associated with high mortality. Using data from a population-based surveillance program, we describe risk factors associated with death in patients with IMI.

Demographic information, total cohort of patients with IMI, N = 236

**Figure:**
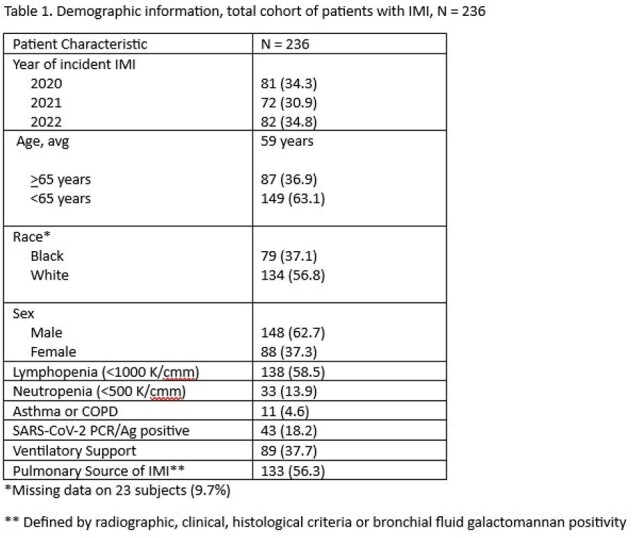

**Methods:**

The Georgia Emerging Infections Program conducted a CDC-funded active IMI surveillance program at 3 academic hospitals in the Atlanta metropolitan area from 2020-2022. Cases were determined based on EORTC/MSG criteria using supportive culture and *Aspergillus* galactomannan (serum or BAL) data. Cases were also ascertained if the treating physician provided a diagnosis of IMI and the patient received mold-active antifungal therapy. Variables listed are for 30 days from incident mold specimen identified and 90 days for COVID-19 diagnosis. Descriptive and univariate analyses evaluated risk factors for 90-day mortality after incident mold specimen identified

Risk Factors for Death from Invasive Mold Infection, Univariate Analysis, N = 236

**Figure:**
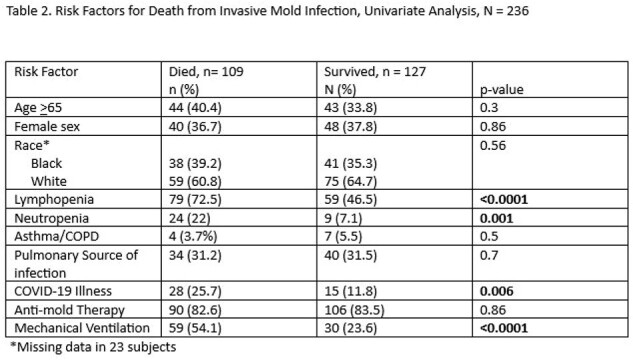

**Results:**

Of 236 patients identified with IMI, average age was 59 years, 134 were White race (56.8%), and 148 (62.7%) were men (Table 1). 109 patients (46.2%) died. Lymphopenia and neutropenia were present in 138 (58%) and 33 patients (13.9%), respectively. SARS-CoV-2 virus infection was found in 43 patients (18.2%). Ventilatory support was used in 89 patients (37.7%), and 133 (56.3%) patients had pulmonary source of IMI (Table 1). The most common identified fungal pathogen was *Aspergillus fumigatus* (18.6%) followed by other unidentified *Aspergillus spp* (6.4%), *Fusarium* (5%), and *Scedosporium* spp (5%); GM was positive in 92 patients (39%).

Univariate analysis revealed significant association with death in patients with lymphopenia (p = < 0.0001), neutropenia (p = 0.001), ventilator use (p < .0001), and COVID-19 illness (p < .006). Factors not significant for death included age >65 years, race, sex, presence of chronic lung disease, pulmonary source of IMI, and receipt of anti-mold therapy (Table 2).

**Conclusion:**

Among patients with IMI in these 3 Atlanta hospitals from 2020-2022 , death was associated with underlying host factors such as lymphopenia and neutropenia status. Similarly, ventilatory support and COVID-19 illness were also associated with mortality. Further studies will examine these associations and differences between mold infections.

**Disclosures:**

All Authors: No reported disclosures

